# Surgical navigation system for temporomandibular joint ankylosis in a child: a case report

**DOI:** 10.1186/s13256-021-03020-z

**Published:** 2021-09-10

**Authors:** Ryo Miyazaki, Akinori Iwasaki, Fumi Nakai, Minoru Miyake

**Affiliations:** 1 Department of Oral and Maxillofacial Surgery, Faculty of Medicine, Kagawa University, 1750-1 Ikenobe, Miki-cho, Kita-gun, Kagawa, 761-0793 Japan; 2 Department of Oral and Maxillofacial Surgery, Kinan Hospital, 46-70, Shinjo-cho, Tanabe-shi, Wakayama, 646-8588 Japan

**Keywords:** Computer-assisted surgical navigation, Temporomandibular joint ankylosis, Child, Pediatric

## Abstract

**Background:**

Computer-assisted surgical navigation systems were initially introduced for use in neurosurgery and have been applied in craniomaxillofacial surgery for 20 years. The anatomy of the oral and maxillofacial region is relatively complicated and includes critical contiguous organs. A surgical navigation system makes it possible to achieve real-time positioning during surgery and to transfer the preoperative design to the actual operation. Temporomandibular joint ankylosis limits the mouth opening, deforms the face, and causes an increase in dental caries. Although early surgical treatment is recommended, there is controversy regarding the optimal surgical technique. In addition, pediatric treatment is difficult because in children the skull is not as wide as it is in adults. There are few reports of pediatric temporomandibular joint ankylosis surgery performed with a navigation system.

**Case presentation:**

A 7-year-old Japanese girl presented severe restriction of the opening and lateral movement of her mouth due to a temporomandibular joint bruise experienced 2 years earlier. Computed tomography and magnetic resonance imaging demonstrated left condyle deformation, disappearance of the joint cavity, and a 0.7-mm skull width. We diagnosed left temporomandibular joint ankylosis and performed a temporomandibular joint ankylosis arthroplasty using a surgical navigation system in order to avoid damage to the patient's brain. A preauricular incision was applied, and interpositional gap arthroplasty with temporal muscle was performed. After the surgery, the maximum aperture was 38 mm, and the limitation of the lateral movement was eliminated.

**Conclusions:**

A navigation system is helpful for confirming the exact target locations and ensuring safe surgery. In our patient's case, pediatric temporomandibular joint ankylosis surgery was performed using a navigation system without complications.

## Background

Temporomandibular joint ankylosis (TMJa) is characterized by immobility of the temporomandibular joint together with the formation of an osseous, fibrous, or fibro-osseous mass fused to the base of the skull. TMJa is commonly caused by trauma, local or systemic infection, or systemic disease such as ankylosing spondylitis, rheumatoid arthritis, or psoriasis, and it may also arise after TMJ surgery [1]. TMJa may induce oral dysfunction and, especially during the growth stage, may cause deformities of the mandible and maxilla. TMJa in children is uncommon, and it is especially challenging for oral surgeons not only because of the technical aspects of the surgery but also because of the difficulty of predicting any impact of the surgery on the patient’s growth [2]. Regardless of the type of surgery selected for TMJa, the first step in the surgical treatment of TMJa is an extended resection of the ankylosed bone. However, the removal of a sufficient amount of ankylosed bone is extremely difficult and highly risky [3]. In addition, bone adhesion further complicates the anatomical structure of the TMJ.

Recent technological advantages have contributed significantly to surgical outcomes. For example, improved navigation systems can accurately indicate critical anatomical structures and identify the safest way to approach the target and the best orientation for safely performing surgery [4]. TMJ surgery carries a risk of brain damage, but computer-assisted navigation systems have recently been reported to be useful guides for complicated oral and maxillofacial surgeries [5]. Real-time intraoperative positioning can be tracked with such a navigation system, and therefore correlations between the preoperative design and the intraoperatively encountered anatomy can be assessed. We here report our use of a computer-assisted navigation system in TMJa surgery in a 7-year-old girl.

## Case presentation

**Fig. 1:**
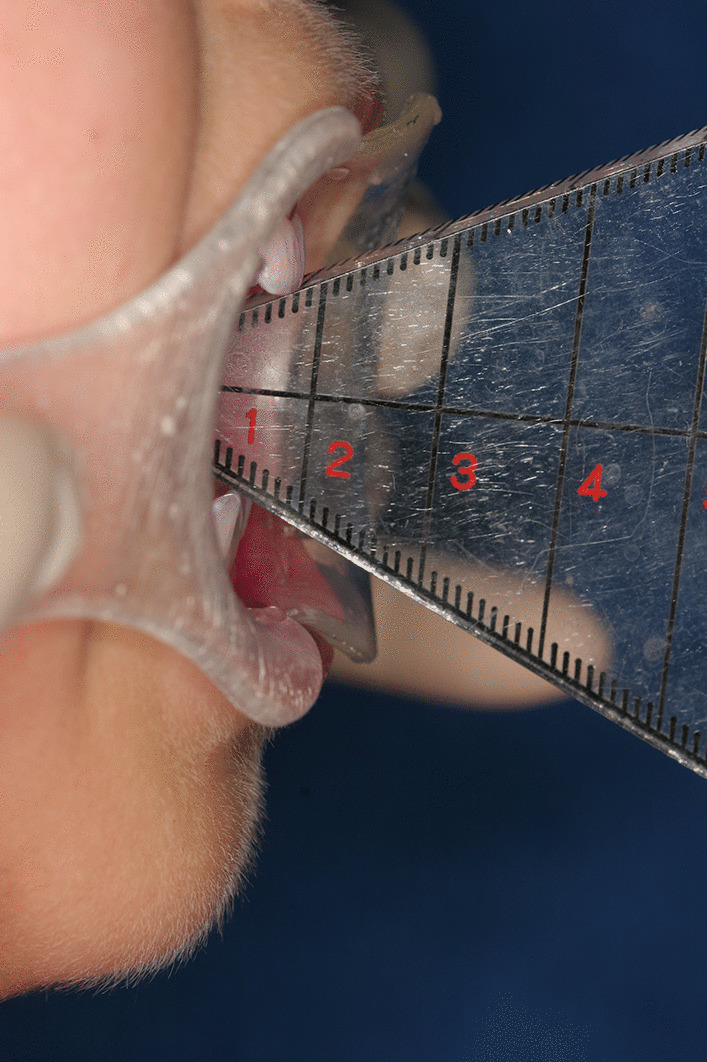
Oral preoperative photo revealing severely limited maximal mouth opening at 13 mm

**Fig. 2:**
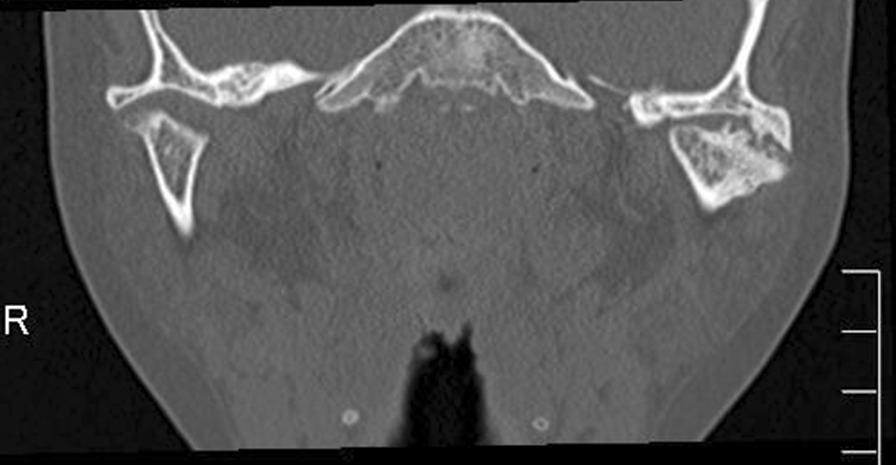
Preoperative computed tomography demonstrated that the mandibular condyle and glenoid fossa were almost osteoadhesive

In 2012, a 7-year-old Japanese girl was referred to our hospital owing to difficulty opening her mouth following a facial bruise caused by a fall from a pull-up bar that occurred in August 2010. Initially, she underwent observation, and gradually her mouth-opening became more restricted. At her first visit, her maximum aperture was 13 mm and the movement of the right mandible was severely restricted (Fig. 1). The opening mainly involved a rolling movement, and no gliding was observed. There were no special medical, family, or psychosocial histories related to this patient. Her panoramic radiography and computed tomography (CT) scans demonstrated left condyle deformation caused by bone addition as well as a severe loss of joint space (Fig. 2). On magnetic resonance imaging, the joint cavity and articular disk were not visible. Based on these findings, we diagnosed left TMJa.

**Fig. 3:**
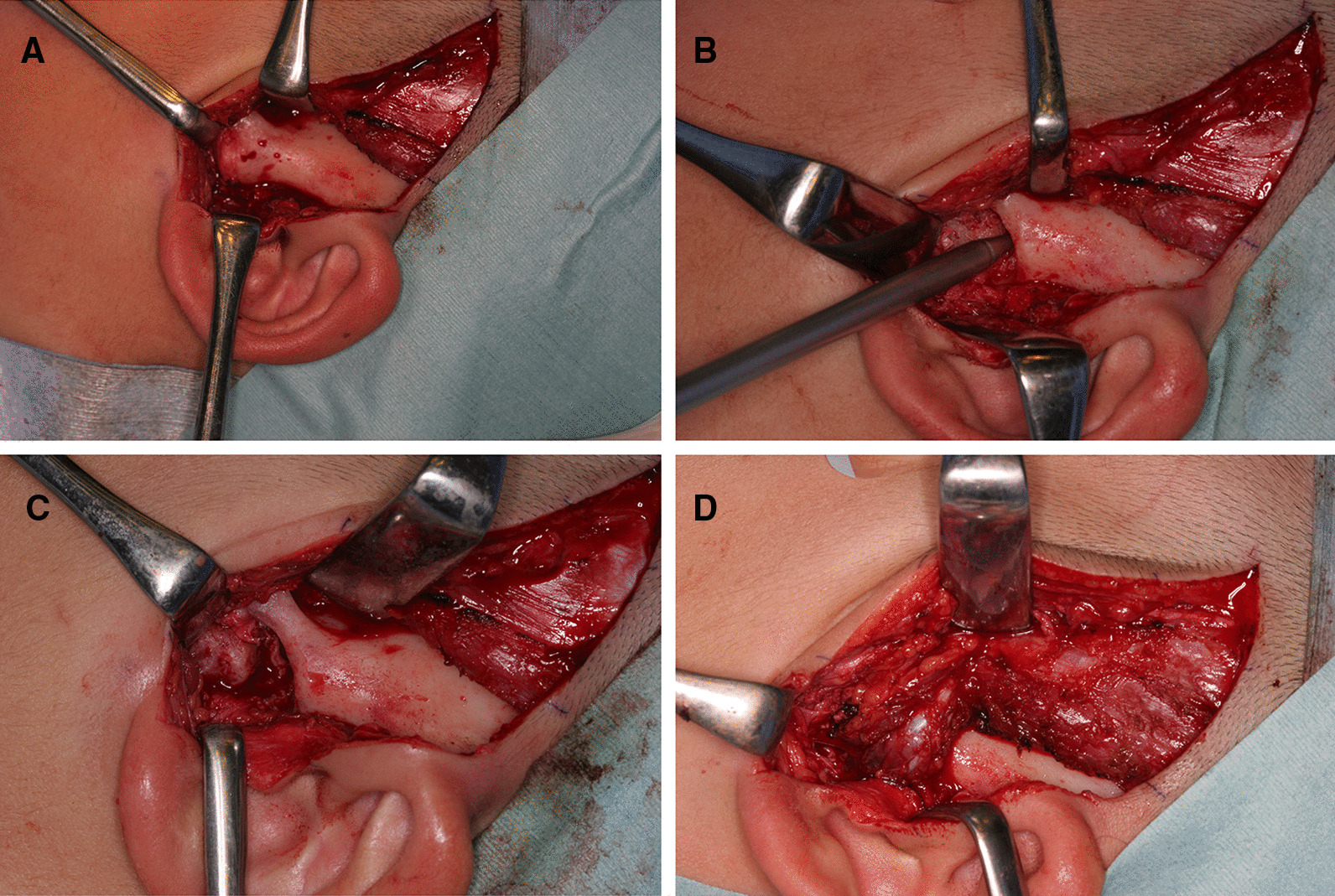
Intraoperative photos demonstrating a bony fusion between the condylar head and glenoid fossa (**A**), the use of the navigation system to confirm the operating position (**B**), the formed articular cavity (**C**), and the temporal muscle insertion into the joint space created by the surgery (**D**)

**Fig. 4.**
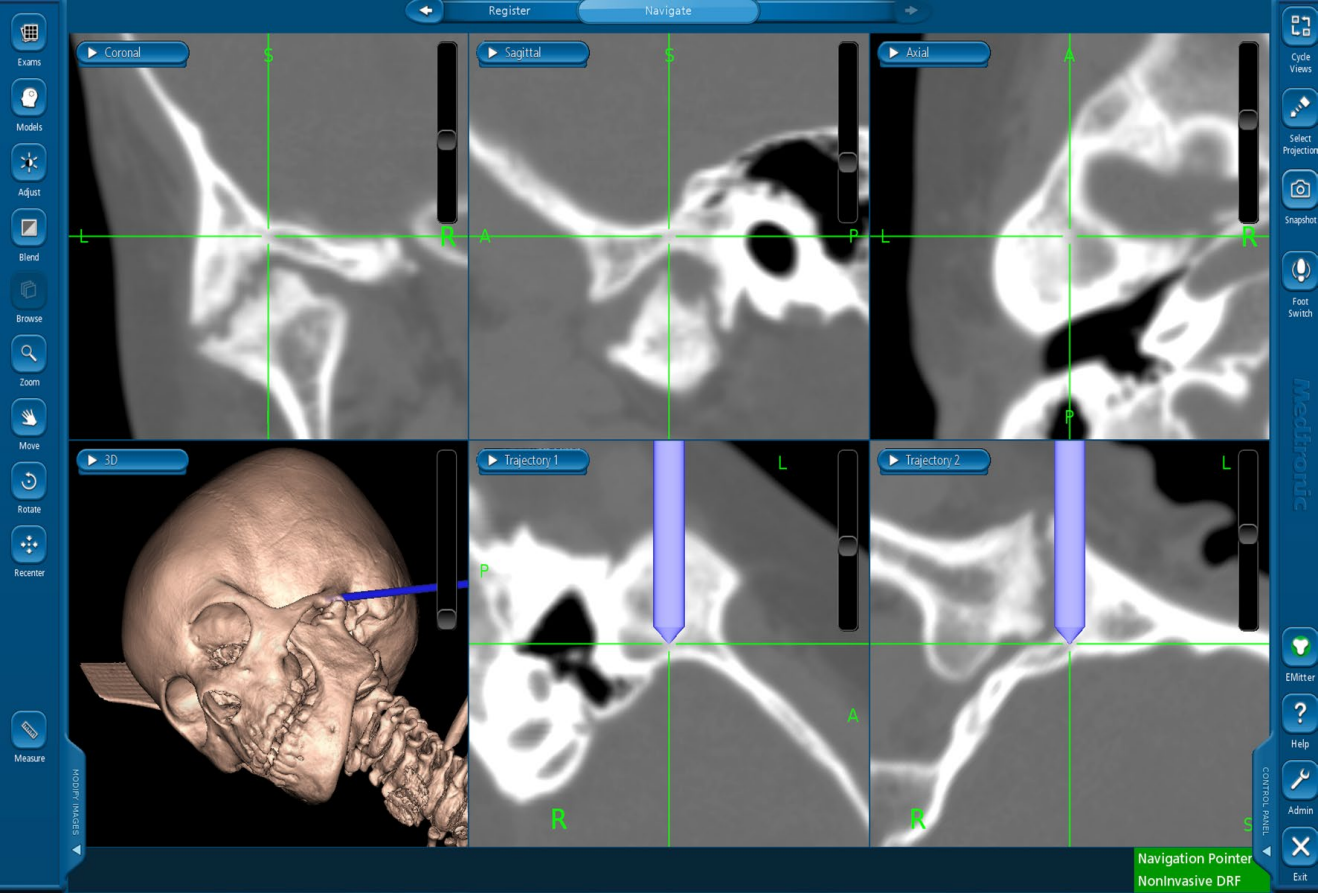
Display of the Medtronic StealthStation S7 workstation

Taking the risk of jaw undergrowth into consideration while also taking care to avoid the risk of brain damage, we performed TMJ arthroplasty using a surgical navigation system (Figs. 3, 4). First, a preauricular incision was made to reveal the TMJa region. Preoperative CT indicated that the skull width was only 0.7 mm at the thinnest point, and the error of the navigation system was confirmed to be 0.3 mm. Therefore, after confirming the position of the medial cranial fossa and the distance from the glenoid fossa to the skull base with the assistance of the Medtronic StealthStation S7 workstation with Synergy Fusion Cranial software (Medtronic Navigation, Louisville, CO), we performed a 10-mm-wide osteotomy and TMJ release. At that stage, the maximum aperture was 32 mm. Finally, the temporal muscle and fascia were inserted into the glenoid fossa created by the surgery. No complications occurred.

**Fig. 5.**
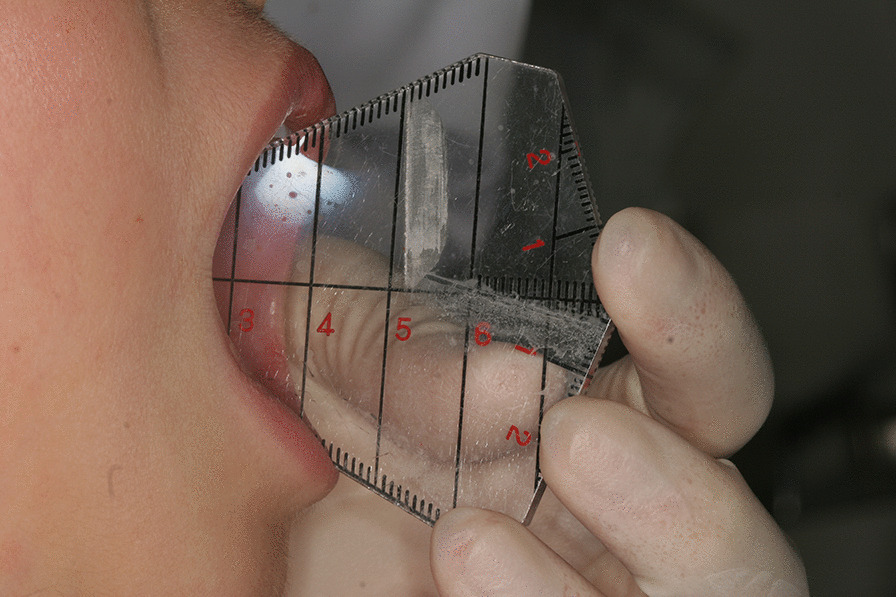
Oral postoperative facial photo revealing a maximal aperture of 38 mm

**Fig. 6.**
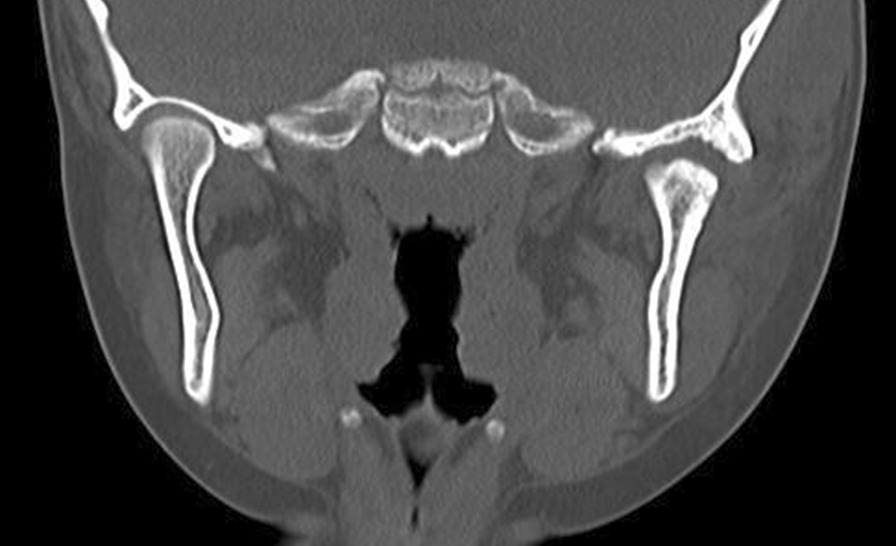
Postoperative CT demonstrating the structure of the patient’s joint cavity

Mouth-opening training was initiated the day after surgery. Six months after surgery, the maximum aperture was 38 mm and there were no longer any impediments to side mandibular movement (Fig. 5). In addition, CT demonstrated a loss of bony adhesions in the condyle and glenoid, improvement of the condylar deformity, and the joint cavity intervention (Fig. 6).

## Discussion

The goal of treatment and early surgical intervention for TMJa is to restore joint function, improve the patient's aesthetic appearance and quality of life, and prevent any recurrence and growth disturbance [6]. Over the last few decades, a number of surgical methods for treating TMJa have been developed, including gap arthroplasty (GA), interpositional gap arthroplasty (IGA), reconstruction arthroplasty, and distraction osteogenesis [7, 8]. However, controversy remains regarding the ideal treatment choices and materials. A recent study found that there was no significant difference in the 24-month recurrence rate between GA and IGA, but that after 24 months significantly fewer recurrence events were seen in patients who underwent IGA compared with those who underwent GA [9]. Although various autogenous and alloplastic materials are used in an IGA, alloplastic materials may induce heterogeneity. In addition, the recurrence rate is significantly higher in patients who underwent IGA using alloplastic materials than in those who underwent IGA using autogenous materials [9]. The temporal muscle is the most commonly used interpositional material because the procedure is convenient and there is little or no risk of heterogeneity.

Computer-assisted surgical navigation systems were initially introduced for use in neurosurgery and have been applied in craniomaxillofacial surgery for 20 years [5, 10]. The first use of a navigation system for TMJa was reported in 2002; the system was found to improve the safety of the operation and to reduce the incidence of complications [11]. Compared with non-navigation surgery, navigation-assisted surgery has shown a significant difference in the lowest thickness of the postoperative skull base, demonstrating that the joint ankylosis procedure could achieve a more extensive removal of ankylosed bone with navigation surgery [3]. Navigation systems also help the surgeon to control the amount of bone removed [3]. In addition, navigation systems are reported to improve quality and reduce risk in skull base surgeries [12]. The skull base is stable, and higher precision can be obtained compared with other neurosurgeries [13]. Therefore, navigation systems can contribute to the accuracy of TMJ surgeries and can minimize their invasiveness.

There are two types of navigation systems: optical and electromagnetic. In the present patient’s case, we used an electromagnetic system because no head fixation was necessary. First, a reference point was set on the patient's forehead, and a magnetic field generator was placed on the side of her head. Registration was then performed with the tracer probe, marker-free. During the operation, the error was confirmed to be 0.3 mm. During the osteotomy, the position of the bone removal site was confirmed by the navigation system, and the surrounding tissue was preserved.

The navigation system used to treat the TMJa in the present pediatric patient helped confirm the exact location of the surgery and helped us perform a safe operation without complications. There have been few reports to date of pediatric TMJa surgery performed using a navigation system. This method has certain disadvantages such as the error value and setting time, and our case required setting time after the general anesthesia had been started. However, our present case indicates that, if the surgery is performed with these points in mind, this method can be beneficial in the treatment of pediatric TMJa.

## Conclusion

We report a case of pediatric TMJa that was treated surgically using a navigation system. The surgery was performed safely, and there was no damage to nearby vital structures. The use of navigation systems in pediatric TMJ surgeries is beneficial.

## Data Availability

Not applicable.
